# Impact of *Solidago virgaurea* Extract on Biofilm Formation for ESBL-*Pseudomonas aeruginosa*: An *In Vitro* Model Study

**DOI:** 10.3390/ph16101383

**Published:** 2023-09-29

**Authors:** Ali Hazim Abdulkareem, Anmar Kamil Alalwani, Mohammed Mukhles Ahmed, Safaa Abed Latef Al-Meani, Mohammed Salih Al-Janaby, Al-Moghira Khairi Al-Qaysi, Ali Ibrahim Edan, Hasan Falah Lahij

**Affiliations:** 1 Department of Biotechnology, College of Science, University of Anbar, Ramadi 31001, Iraq; Ali.hazim@uoanbar.edu.iq (A.H.A.); anmar_kamil@uoanbar.edu.iq (A.K.A.); sc.safaa-meani@uoanbar.edu.iq (S.A.L.A.-M.); mohammed.salih.hussein1@gmail.com (M.S.A.-J.); mqaysi89@uoanbar.edu.iq (A.-M.K.A.-Q.); 2 Medical Laboratory Technology, Al-Huda University College, Ramadi 31001, Iraq; ali.ibrahem@uoalhuda.edu.iq; 3 Medical Laboratory Technology, Almaarif University College, Ramadi 31001, Iraq; hassan.falah@uoa.edu.iq

**Keywords:** *Solidago*, ESBLs, MDR, Biofilm, *Pseudomonas aeruginosa*, Antimicrobial resistance

## Abstract

The increasing disparity between antimicrobial resistance (AMR) and the development of new antimicrobials continues to pose a significant global health concern. However, plant extracts have shown promise in combating this issue either through their inherent antimicrobial activity or by serving as potential reservoirs of effective antimicrobial compounds. These compounds have the ability to target pathogenic biofilms and inhibit the production of extended-spectrum β -lactamases (ESBLs). However, there is limited research available on the antibacterial properties of goldenrod extract. Thus, the objective of this study was to investigate the impact of *S. virgaurea* (SV) extract on the viability and ability to form biofilms of ESBL-*Pseudomonas aeruginosa (P. aeruginosa)*. A cross-sectional study was conducted from August 2022 to March 2023. The broth microdilution method was employed to determine the minimum inhibitory concentration (MIC) of the (SV) extract. Subsequently, the minimum bactericidal concentration (MBC) was determined based on the MIC values obtained. The antibiotic susceptibility of bacteria was evaluated using the Kirby disk diffusion assay and an Antimicrobial Susceptibility Testing (AST) card in conjunction with the Vitek-2 compact system. Biofilm formation was evaluated using Congo red and a 96-well Elisa plate, while the presence of extended-spectrum β-lactamases (ESBLs) was estimated by measuring the reduction of nitrocefin at a wavelength of 390 nm. In addition, treatment of biofilm and ESBL activity with SV extract using 96-well Elisa plate and nitrocefin hydrolyzing, respectively. The resistance rates of *P. aeruginosa* isolates to the tested antibiotics were as follows: Levofloxacin 33%, Ciprofloxacin 40%, Amikacin 49%, Meropenem 50%, Cefepime 70%, Ceftazidime 75%, Cefotaxime 85%, Piperacillin-Tazobactam 90%, Amoxiclav 97%, Ampicillin 99%, Ceftriaxone 100%. The prevalence of MDR-*P. aeruginosa*, XDR-*P. aeruginosa*, PDR-*P. aeruginosa* and non-MDR-PA were 40% (*n* = 40), 7% (*n* = 7), 3% (*n* = 3) and 50% (*n* = 50), respectively. From the GC–MS results, it was observed that the presence of Octadecane, Clioquinol, Glycerol tricaprylate, hexadecanoic acid, cis-13-octadecenoic acid, oleic acid and Propanamide were the major components in the *Solidago* extract. In the determination of plant crude extracts, the values ranged between 0.25 and 64 mg/mL against bacteria. The resulting activity of the extract showed a significant statistical relationship at a *p*-value ≤ 0.01 against ESBL production and biofilm formation in *P. aeruginosa*. The *S. virgaurea* extract exhibited effectiveness in inhibiting biofilm formation and combating *P. aeruginosa* strains that produce extended-spectrum β-lactamases (ESBLs).

## 1. Introduction

**Figure 1:**
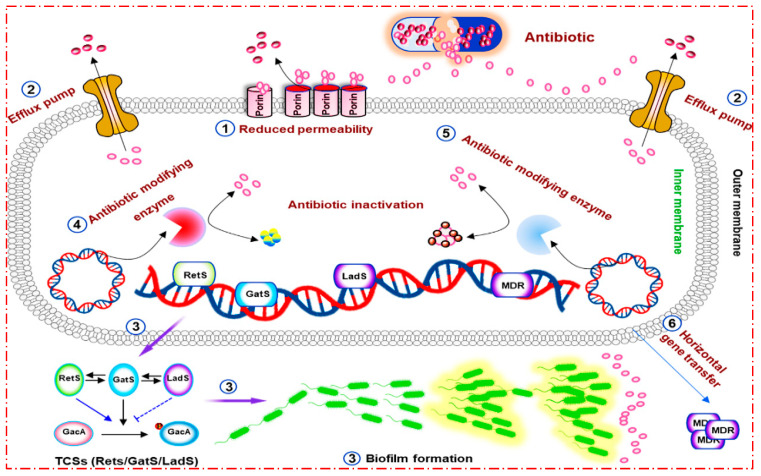
Mechanisms of antibiotic resistance in *P. aeruginosa*. Reprinted/adapted with permission from Ref. [5]. 2022, Qin, S.; Xiao, W.; Zhou, C.; Pu, Q.; Deng, X.; Lan, L.; Liang, H.; Song, X.; Wu, M.

*Pseudomonas aeruginosa (P. aeruginosa)* is an opportunistic pathogen under the pseudomonadaceae family. The characteristics of this bacterium are a rod shape under a microscope, Gram-negativity, its possession of a single flagellum [1], its ability to produce different pigments (pyocyanin: blue-green; pyoverdine: yellow-green and fluorescent; pyorubin: red-brown), a tortilla-like odour and its ability to grow at 42 °C that help bacteriologists in differentiating this bacteria from various other *Pseudomonas* species [2]. It has the ability to infect individuals who have cystic fibrosis, burn wounds, weakened immune systems, chronic obstructive pulmonary disorder (COPD), cancer and those who require ventilation due to severe infections, such as COVID-19 [3]. Recently, antimicrobial resistance became a public health problem that threatens communities and that causes increasing high morbidity [4]. *P. aeruginosa* exhibits various mechanisms of antibiotic resistance, which can be categorized into different types. These include intrinsic resistance (such as outer membrane permeability, efflux pumps, antibiotic-modifying enzymes and antibiotic-inactivating enzymes, such as: extended-spectrum β-lactamases (ESBLs)), acquired resistance (resulting from mutations or acquisition of resistance genes) and adaptive resistance (related to biofilm-mediated resistance). Figure 1 illustrates these classifications. Biofilms, facilitated by quorum-sensing signaling molecules, play a role in activating resistance by forming physical barriers that impede the penetration of antibiotics into the bacterial cells [5]. ESBL is an enzyme manufactured by bacteria to confer resistance against extended-spectrum penicillin, cephalosporins and monobactams—except for cephamycins and carbapenems. It is susceptible to inhibition by β-lactamase inhibitors, such as clavulanic acid. A concerning upward trajectory has been documented in the rise of resistance to extended-spectrum cephalosporins due to Enterobacteriaceae that produce ESBLs [6,7].

Due to advancements in scientific knowledge regarding the medicinal attributes of plants, there has been a surge of interest in natural sources of antibiotics. These sources are particularly appealing due to their minimal toxicity, beneficial pharmacological effects and economic feasibility [8,9]. *S. virgaurea (SV)* consisted of many active compounds that lead for using it in the treatment of many diseases, including kidney inflammation, urinary tract infection, cystitis, diarrhea and arthritis [10,11]. *SV* extracts also limit biofilm formation and reduce the biomass of pre-grown *Candida*-bacterial biofilms [12]. *S. virgaurea* extracts contain a diverse range of compounds, including glycosides, such as virgaureoside and leiocarposide, as well as aglycones, such as vanillic acid and gallic acid [13]. The goldenrod herb is often recommended for individuals experiencing urinary system bacterial infections and kidney inflammation due to its diuretic properties [8,11]. In fact, there are no reports describing antimicrobial activities of goldenrod herb in Iraq. Therefore, this study aimed to determine antipseudomonal activity using a conventional method (well-agar diffusion method), estimate Sub-MIC of goldenrod herb using resazurine–colored method, investigate activity of goldenrod herb to inhibit biofilm formation of *P. aeruginosa* and evaluate activity of goldenrod herb against ESBL-producing *P. aeruginosa*.

## 2. Results

### 2.1. Distribution of P. aeruginosa According to Source

**Figure 2:**
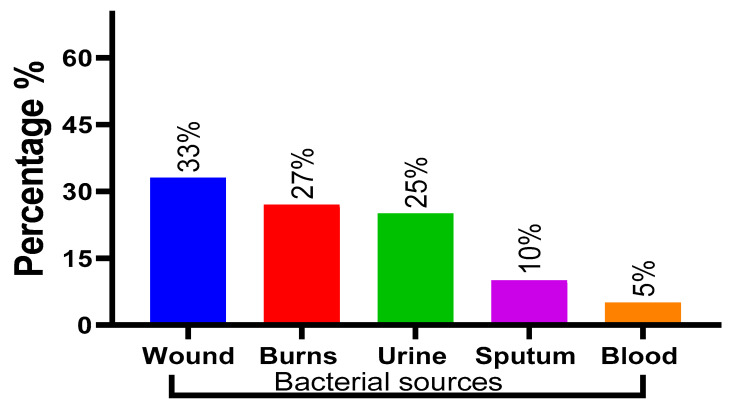
Distribution of *P. aeruginosa* based on source.

A total of 100 unique *P. aeruginosa* isolates were collected from patients, with 66 (66%) originating from male patients and 54 (54%) from female patients. An analysis of the distribution of *P. aeruginosa* in clinical samples revealed that the majority of isolates (33%) were obtained from wounds, followed by burns (27%), urine (25%), sputum (10%) and blood (5%), as depicted in Figure 2.

### 2.2. Identification of P. aeruginosa

To confirm the diagnosis of *P. aeruginosa*, the bacterial isolates were initially diagnosed as *P. aeruginosa*. The bacterial isolates were cultured on blood agar, MacConkey agar, cetrimide agar and chromogenic pseudomonas agar under aerobic conditions followed by other diagnostic tests as shown Table 1.

### 2.3. Susceptibility Test

**Figure 3:**
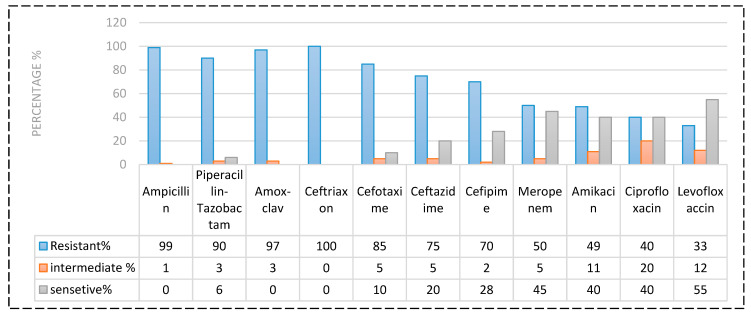
Percentage of antibiotic resistant *P. aeruginosa*.

**Figure 4:**
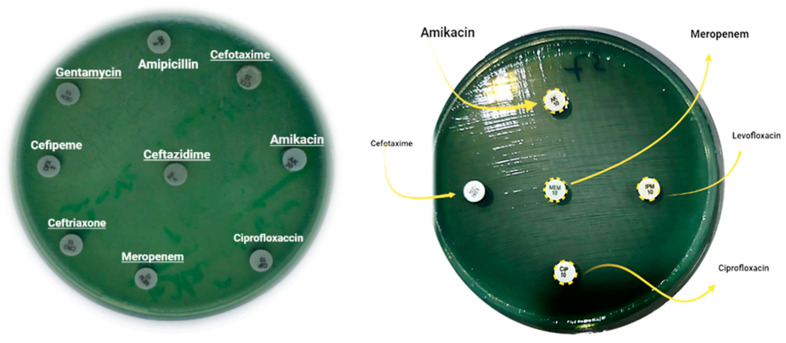
Kirby–Bauer Disk Diffusion Susceptibility Test for antibiotics in *P. aeruginosa*. No inhibition zone: Full resistance; Greenish-colored: producing of pyocyanin pigment by *P. aeruginosa*.

Based on the CLSI interpretive criteria [14], resistance rate among *P. aeruginosa* isolates to antibiotics tested was as follow (Figure 3 and Figure 4): Levofloxacin 33% (*n* = 33), Ciprofloxacin 40% (*n* = 40), Amikacin 49% (*n* = 49), Meropenem 50% (*n* = 50), Cefipime70% (*n* = 70), Ceftazidime75% (*n* = 75), Cefotaxime 85% (*n* = 85), Piperacillin-Tazobactam 90 (*n* = 90), Amoxiclav 97% (*n* = 14), Ampicillin 99% (*n* = 99), Ceftriaxone 100% (*n* = 100). The prevalence of MDR-*P. aeruginosa*, XDR-*P. aeruginosa*, PDR-*P. aeruginosa* and non-MDR-PA was 40% (*n* = 40), 7% (*n* = 7), 3% (*n* = 3) and 50% (*n* = 50), respectively.

### 2.4. Qualitative and Quantitative Detection of Biofilm Forming–P. aeruginosa

**Figure 5:**
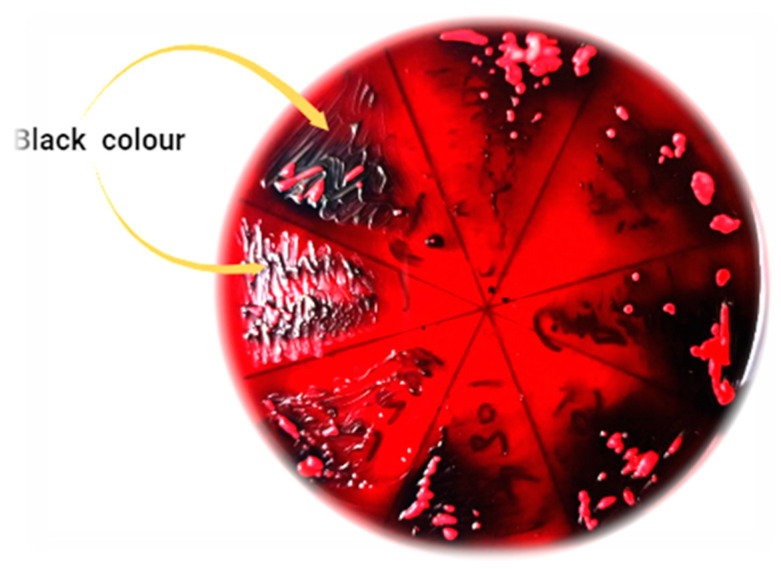
Congo red method to detect biofilm formation in *P. aeruginosa*. Black colonies as positive for biofilm.

The qualitative test for biofilm formation showed that among the tested isolates, both strains of *P. aeruginosa* produced black colored colonies on the CRA with glucose as shown in Figure 5. In the biofilm formation method, we selected 50 isolates to test forming biofilm. Out of the total isolates, 80% (*n* = 40) exhibited biofilm phenotypes, which were categorized as follows: 40% (*n* = 20) demonstrated strong biofilm production, 24% (*n* = 12) showed moderate biofilm production, 16% (*n* = 8) exhibited weak biofilm production and 20% (*n* = 10) were identified as non-biofilm producers.

### 2.5. Identification of Active Compounds of Goldenrod Herba

**Figure 6:**
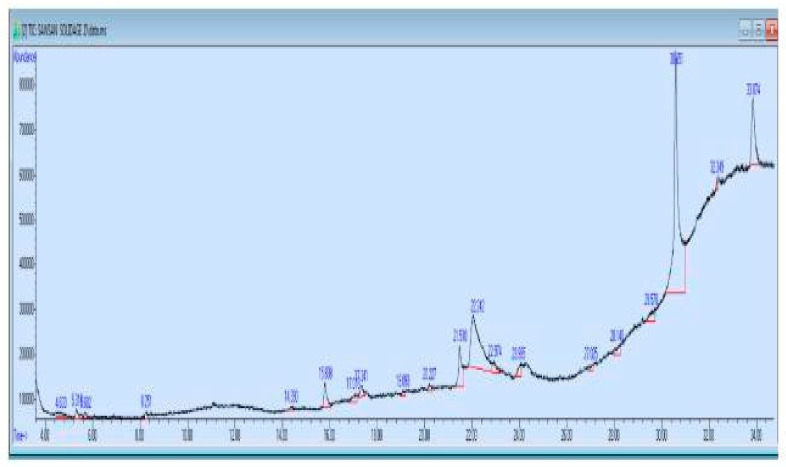
GC–MS analysis of SV extract.

An analysis of the golden rod extract by GC–MC was conducted to determine the active compounds found in this extract as shown in figure and Table 2, Figure 6.

GC–MS chromatogram of *Solidago virgaurea* extract showed twenty major peaks (Figure 5) and have been identified after comparison of the mass spectra with NIST library (Table 2), indicating the presence of twenty phytocomponents. From the results, it was observed that presence of Octadecane, Clioquinol, Glycerol tricaprylate, hexadecanoic acid, cis-13-octadecenoic acid, oleic acid and Propanamide were the major components in the extract.

### 2.6. Determination of MIC for Antimicrobial Agents against P. aeruginosa

The concentration range of MIC values for the tested antibiotics was 8 to 128 µg/mL. All five strains of *P. aeruginosa* (P1 to P3) exhibited high MIC values for ceftazidime, ranging from 16 to 128 µg/mL. On the other hand, the SV extracts demonstrated a diverse pattern of MIC values against the bacterial isolates, ranging from 0.25 to 64 mg/mL, as shown in Table 3.

### 2.7. Detection of ESBL Activity and Biofilm Formation in P. aueroginosa

**Figure 7:**
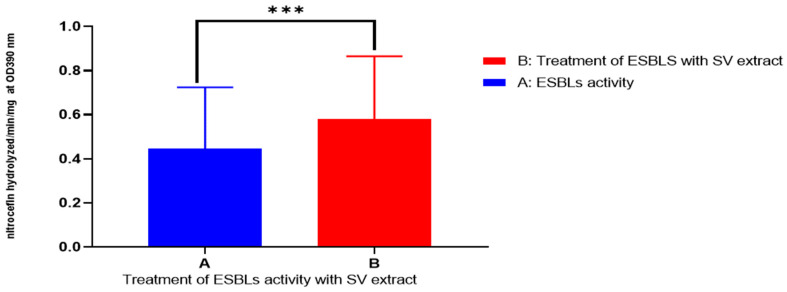
Effect of goldenrod herb against ESBL producing P. aeruginosa. ***: strong significant.

**Figure 8:**
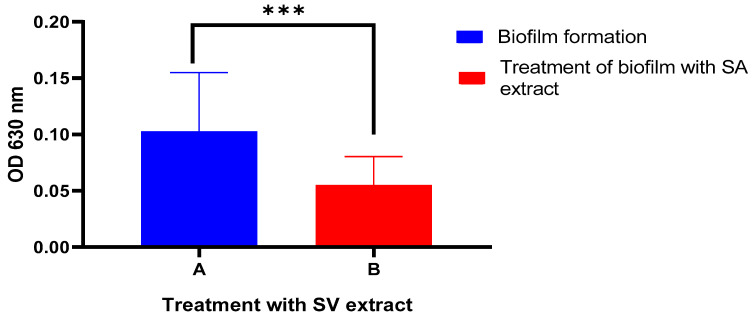
Anti-biofilm producing *P. aeruginosa.* ***: strong significant.

As shown in Figure 7 and Figure 8 and Table 4, results showed that Solidago inhibited ESBL production and biofilm formation in *P. aeruginosa* significant statistical relationship at *p*-value ≤ 0.01. In Table 4, the average level of biofilm formation (M ± SD: 0.1028 ± 0.05215 (nitrocefin hydrolyzed/min/mg)) showed a reduction upon the addition of the extract, decreasing to (M ± SD: 0.05515 ± 0.02532). Conversely, the hydrolysis of ESBLs increased in the presence of the extract, rising from (M ± SD: 0.4447 ± 0.2793) to (M ± SD: 0.5812 ± 0.2837).

### 2.8. Association of ESBL Production and Biofilm Formation among P. aueroginosa Isolates

According to the findings presented in Table 5, ESBL-producing strains of *P. aeruginosa* exhibited significantly higher biofilm formation capability compared to ESBL non-producing strains (*p* < 0.05). Among the 36 ESBL-producing *P. aeruginosa* strains, 20 (55.5%) were classified as strong biofilm producers, 8 (22.8%) as moderate biofilm producers, 2 (5.5%) as weak biofilm producers and 6 (16.6%) as non-biofilm producers. In contrast, among the 14 ESBL non-producing *E. coli* strains, 5 (35.7%) were strong biofilm producers, 2 (14.4%) were moderate biofilm producers, 3 (21.4%) were weak biofilm producers and 4 (28.5%) were non-biofilm producers.

### 2.9. Synergistic Effects between Some Antibiotics and SV Extracts against P. aueroginosa

Table 6 shows no synergistic effects between SV extract with Ceftazidime, Cefepime and Amikacin.

## 3. Discussion

The emergence of antimicrobial resistance in *P. aeruginosa* poses a significant challenge in effectively controlling infections caused by this pathogen [14]. The comprehensive surveillance conducted in European countries for 2017 revealed a wide spectrum of combined resistance to antimicrobial agents, including piperacillin ± tazobactam, ceftazidime, fluoroquinolones, aminoglycosides and carbapenems. The prevalence of combined resistance varied significantly, ranging from 0% in Iceland to 59.1% in Romania [15]. In Iran, the estimated prevalence of multidrug-resistant (MDR) *P. aeruginosa* has been reported at 58%, with variations observed across different geographical areas. The highest rate was observed in Tehran, with a prevalence of 100%, while the lowest rate was recorded in Zahedan at 16% [16]. In a recent study conducted by Bavasheh et al. [17], it was discovered that 27.8% of clinical *P. aeruginosa* isolates exhibited multidrug resistance (MDR). Furthermore, the prevalence of isolates demonstrating resistance to at least three antimicrobial groups in our study was found to be 20%, which was lower compared to findings reported in other studies [18]. While the rate of multi-resistance in the current study was comparatively low, it is still a cause for concern as it indicates a potential threat that restricts treatment options in the therapeutic centers under investigation.

A study done by [19], reported that propenamide was recognized for its ability to display antimicrobial and antiviral properties. In another investigation conducted by Sachin Chaudhary (2019), it was observed that propenamide, when administered at a concentration of 50 µg/mL, demonstrated the highest level of efficacy against Gram-negative bacterial strains. Notably, these strains included *Escherichia coli* and *Pseudomonas aeruginosa*, outperforming the effects of ciprofloxacin [20]. Yasir et al. (2017) reported that propenamide exhibited robust inhibition against *Bacillus subtilis* and *Escherichia coli*, displaying zone of inhibition measurements of 16 mm for both bacterial strains [21].

In fact, A study by [22] indicated that some alkanes, include Octadecane have a good antimicrobial effect especially on *Staphylococcus aureus* and *Escherichia coli*.

A search across the Pubmed, Embase, and Web of Science databases yielded six articles related to the antibacterial activity of clioquinol. This makes it the second most frequently reported activity for the substance in the literature. Unlike studies on clioquinol as an antifungal agent, the research conducted on its antibacterial activity primarily focuses on specific bacterial species. These studies often explore mechanisms of bacterial resistance to clioquinol, such as the investigation conducted by Blanco et al. (2018) [23]. In their study, the authors investigated the impact of clioquinol on the expression of the smeVWX gene, which is responsible for efflux pumps and resistance mechanisms in *Stenotrophomonas maltophilia*. Their findings led to the conclusion that clioquinol induces selective mechanisms of bacterial resistance. Specifically, in the studied strain PBT02 of *S. maltophilia*, clioquinol and pronamide were found to a potent against antibiotic resistant bacteria. The authors employed the Biolog Phenotype Microarrays technology as their chosen methodology. A similar trial conducted by Majumdar et al. also explored related aspects. In their investigation, the researchers focused on the *rarA* gene found in *Klebsiella pneumoniae*, which is responsible for various resistance mechanisms against multiple antimicrobial compounds. The experimental results indicated that the *rarA* gene plays a role in enhancing resistance to clioquinol in host bacteria by increasing the expression of nitric oxide synthase. Notably, nitric oxide serves as an intrinsic modulator, dampening the pharmacological effects of clioquinol [24].

Studies have demonstrated that Solidago extract effectively hinders the development of biofilms in various bacteria, such as *Escherichia coli* and *Staphylococcus aureus*. Biofilms are intricate bacterial structures deeply embedded within a matrix of extracellular slime. They frequently exhibit resistance to antibiotics and other antimicrobial substances, posing challenges for treatment [25].

Solidago extract seems to hinder the formation of biofilms by impeding bacterial adhesion to surfaces and by disturbing the extracellular matrix. In one investigation, Solidago extract demonstrated the capability to decrease *E. coli* adhesion to plastic surfaces by as much as 80%. In a separate study, Solidago extract was observed to disrupt the extracellular matrix within *S. aureus* biofilms, resulting in the detachment of bacteria from the biofilm structure [26].

The precise mechanisms through which Solidago extract hinders biofilm formation remain incompletely elucidated, but it is believed to entail the inhibition of various enzymes and proteins pivotal in the process of biofilm formation. More extensive research is required to gain a comprehensive understanding of Solidago extract’s modes of action and to evaluate its potential as a therapeutic option for infections associated with biofilms.

There is some indication that Solidago extract might impede the functioning of extended-spectrum β-lactamases (ESBLs). ESBLs are enzymes produced by certain bacteria that can render beta-lactam antibiotics ineffective against infections. However, there is no scientific substantiation to support the notion that Solidago extract has a synergistic effect when used in combination with antibiotics. In fact, some research studies have revealed that Solidago extract can potentially interfere with the effectiveness of antibiotics. For instance, a study conducted by Natasha et al. in 2018 discovered that Solidago extract hindered the activity of the antibiotic ampicillin against *Streptococcus* spp., a type of bacteria responsible for infections in humans. Additionally, the study determined that Solidago extract did not possess any inherent antibacterial properties [27]. In a separate investigation conducted by Dorota et al. and published in 2019, it was observed that Solidago extract impeded the efficacy of the antibiotic ciprofloxacin when used against *Escherichia coli*, another bacterial strain responsible for human infections. Moreover, this study also revealed that Solidago extract did not exhibit any inherent antibacterial properties on its own [28].

## 4. Materials and Methods

**Figure 9:**
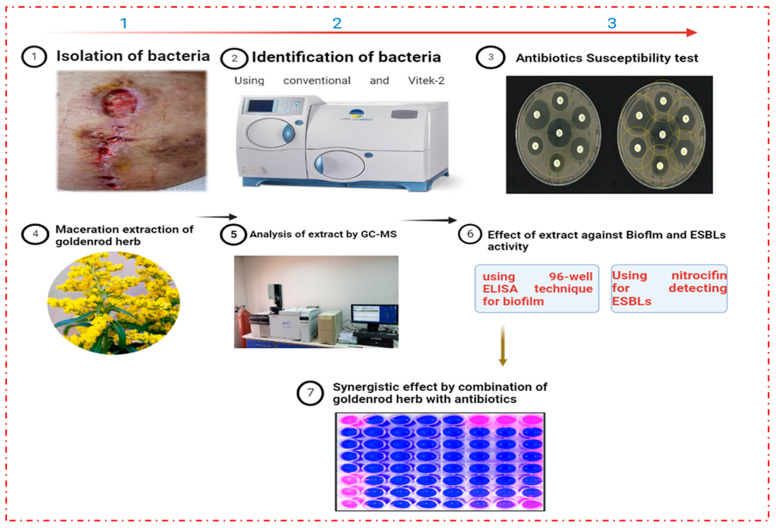
Summary of methods under this study. This image was designed in this study by researcher Mohammed Mukhles Ahmed.

Based on Figure 9, several working methods are summarized in it.

### 4.1. Study Design

The descriptive cross-sectional research took place in the bacteriology unit of General Ramadi Teaching Hospital in Ramadi city, Iraq. The study extended over multiple months, commencing in August 2022 and concluding in March 2023. A total of 435 samples were gathered from diverse clinical sources, encompassing wounds, burns, urine, sputum and blood, all collected under stringent sterilization measures. The inclusion criteria stipulated that samples were taken prior to any antibiotic treatment, maintaining rigorous aseptic conditions and ensuring that the participants were male and aged over 35 years.

### 4.2. Isolation of Bacteria

One hundred of *P. aeruginosa* were isolated from clinically different sources, including wounds, sputum, urine, blood and burns. All isolates were streaked on MacConkey Agar, blood agar that was prepared based on the manufacturer’s instruction of Merck, Germany, then incubated at 42 °C for 24 h.

### 4.3. Identification of Bacterial Isolates

**Figure 10:**
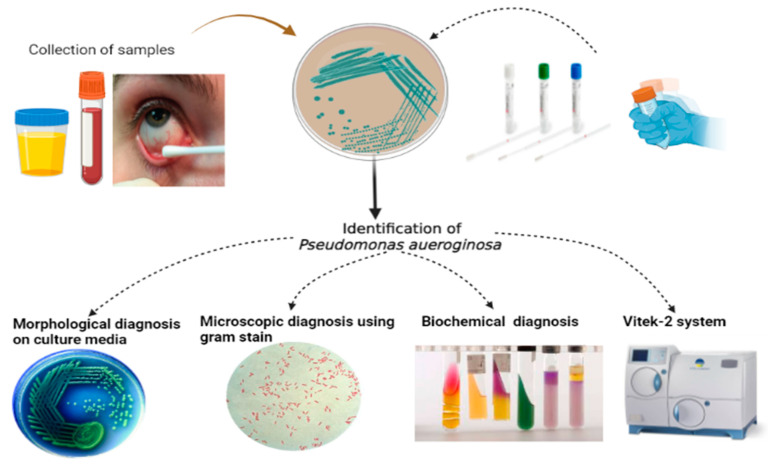
Identification of *P. aueroginosa.* This image was designed in this study by researcher Mohammed Mukhles Ahmed.

According to Figure 10, The bacterial strain was confirmed using the biochemical reactions by *Bergey’s Manual of Systemic bacteriology* and Finegold and Marti [29]. All isolates identified using two methods included 1-conventional methods (on culture media, biochemical test and gram stain) and 2-automated methods using Vitek-2 compact system.

### 4.4. Antibiotics Susceptibility of P. aueroginosa

The profile of this test was investigated based on CLSI [30] and EUCAST guidelines [31] using VITEK 2 compact system (Biomerieux, Craponne, France) in accordance with the manufacturer’s instructions and Kirby–Bauer disk diffusion method [32] for different antibiotics, including Ampicillin, Piperacillin-Tazobactam, Amoxiclav, Ceftriaxone, Cefotaxime, Ceftazidime, Cefepime, Meropenem, Amikacin, Ciprofloxacin and Levofloxacin (Mast Group, Bootle, England).

### 4.5. Collection of Goldenrod Herb

Goldenrod herb was purchased from T&D company (manufactured in Germany). This medicinal herb was identified by Asst. Prof. Dr. Mohammed Othman of the Herbarium, Center Of Desert Studies, University of Anbar (No. 21; Date: 22 September 2022).

### 4.6. Maceration Extraction of Goldenrod Herb

**Figure 11:**
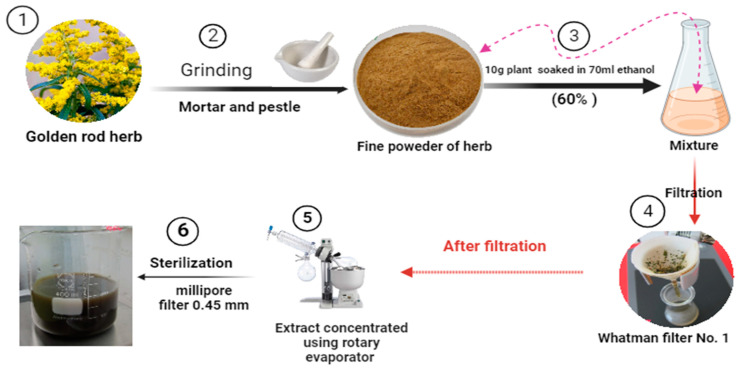
Maceration extraction of golden rod herb. This image was designed in this study by researcher Mohammed Mukhles Ahmed.

As shown in Figure 11, goldenrod herb was extracted with maceration method using a 60% ethanol alcohol as a solvent. Therefore, 10 g of goldenrod herb powder was added to 70 mL of ethanol alcohol. Then, the extraction procedure was duplicated and carried out over a span of 2 days. Subsequently, the resulting extract underwent filtration using a Whatman filter No. 1, followed by concentration under reduced pressure utilizing a rotary evaporator set at 40 °C. The resultant extract solution was then subjected to sterilization by passing it through a Millipore membrane filter with a pore size of 0.45 mm. The dried extracts were subsequently stored at 4 °C for future utilization.

### 4.7. Analysis of Goldenrod Herb Extract Using Gas Chromatography–Mass Spectrometry

According to the Agilent manufacturer’s instructions (Santa Clara, CA, USA), the goldenrod herb extract was analyzed using GC–MS on an Agilent GC–MS instrument (model 7820A) from the USA. The analysis was conducted under the following conditions: an Agilent HP-5ms Ultra Inert analytical column (30 m length × 250 µm diameter × 0.25 µm inside diameter) was used. A volume 1 µL of the extract was injected into the instrument at a pressure of 11.933 psi. The GC inlet line temperature was set at 250 °C, and the auxiliary heaters temperature was set at 310 °C. The carrier gas used was helium with a purity of 99.99% at a constant flow rate of 1 mL/min. The injector temperature was maintained at 250 °C. The scan range for mass spectrometry was set from *m*/*z* 50 to 500. The injection type was set to split mode. The oven program included temperature ramping: Ramp 1 from 60 °C with a hold time of 1 min, Ramp 2 from 60 °C to 180 °C at a rate of 7 °C/min and Ramp 3 from 180 °C to 280 °C at a rate of 7 °C/min. The total analysis time was approximately 33 min.

### 4.8. Determination of Minimum Inhibitory Concentration (MIC) and MBC

The Resazurin microtitre-plate assay (REMA) was utilized to determine the minimum inhibitory concentration (MIC) of antibiotic solutions and natural products with slight modifications. In aseptic conditions, 100 µL of Mueller–Hinton broth (MHB) (Merck, Darmstadt, Germany) containing 1024 µg/mL of ceftriaxone, cefotaxime or natural products (separately) were added to the first row of a 96-well plate, which already contained 100 µL of MHB broth. Then, 100 µL from the first row was transferred to the second row, creating a serial dilution. Subsequently, 10 µL of a bacterial suspension containing 1.5 × 10^8^ CFU/mL was added to each well. The plates were carefully sealed with para-film to prevent dehydration and incubated overnight at 37 °C. After incubation, 15 µL of resazurin solution (Alamar blue) was added to each well, and the plate was further incubated for 1 h to assess color changes. Visual evaluation was performed to determine positive alterations in the resazurin color, indicating a change from purple to pink, red or colorless. The lowest concentration that did not show any change in resazurin color was recorded as the MIC value [33].

### 4.9. Qualitative Detection Based on Congo Red Agar (CRA) Method

Freeman et al. described the Congo red agar (CRA) method, which is a qualitative assay used to detect microorganisms that produce biofilms. This method involves observing a color change in colonies inoculated on CRA medium. The CRA medium was prepared by combining 0.8 g of Congo red (Merck, Germany), 36 g of sucrose (Merck, Germany) and 37 g/L of brain–heart infusion (BHI) agar (Merck, Germany). Following an incubation period of 24 h at 37 °C, the morphology of colonies displaying different colors allowed for differentiation between biofilm producers and non-biofilm producers. Specifically, black colonies with a dry crystalline consistency indicated the presence of biofilm while colonies that retained a pink color were considered non-biofilm producers [34].

### 4.10. Quantitative Detection of Biofilm Based on 96-Well Microtiter Plate

Biofilm formation was assessed in a 96-well polystyrene microtiter plate, following a previously described method with slight modifications [35]. To begin, 10 μL of overnight cultures of *P. aeruginosa* were added to 190 μL of fresh BHI broth supplemented with 32 μg of goldenrod herb extract. As a control, the same volume of BHI broth (Merck, Germany) was used. The plate was then incubated with shaking at 200 rpm for 1 h, followed by a stationary incubation at 37 °C for 18 days. Afterward, the wells were gently washed twice with deionized water to remove any planktonic cells. The remaining biofilm cells were stained with a 1% (*v*/*v*) crystal violet solution (Merck, Germany) for 15 min. Excess dye was washed off with deionized water, and 200 μL of 95% ethanol was added to dissolve the crystal violet stains. The absorbance of the solutions was measured at 630 nm to quantify biofilm formation [36]. Biofilm formation was categorized into four distinct groups based on the following criteria: If the optical density (OD) value was less than ODc, the biofilm formation was considered negative. If the OD value fell between ODc and 2*ODc, the biofilm formation was classified as weak. In the range of 2*ODc to 4*ODc, the biofilm formation was categorized as moderate. Finally, if the OD value exceeded 4xODc, the biofilm formation was characterized as strong.

### 4.11. Antibiofilm and AntiESBL Production of Goldenrod Herb

The β-lactamase activity of *P. aeruginosa* was assessed using UV-Vis Spectrophotometer (Aligent, Santa Clara, CA, USA) by measuring the hydrolysis of nitrocefin (Merck, Germany). The assay mixture consisted of 83 μg of nitrocefin, 167 μg of BSA (Merck, Germany), 10% glycerol(Merck, Germany) and 0.33 mL (0.6 μg/mL of albumin) (Merck, Germany) of cell lysate containing β-lactamase in a final volume of 1.5 mL of 50 mM phosphate buffer. The activity of β-lactamase was monitored by measuring the reduction in absorbance at 390 nm over a 10-min period at 37 °C. Enzyme activity was quantified as μmol of nitrocefin hydrolyzed per minute per milligram of protein, with the calculation based on the molar extinction coefficient of 15,000 M^−1^ cm^−1^ for nitrocefin. As a control, *P. aeruginosa ATCC25922* was used in this method. Additionally, sub-minimum inhibitory concentrations (MIC) of natural products were employed as anti-ESBLs in the study [37].

### 4.12. Combination between Antibiotics and Goldenrod Herb Based on Checkerboard Assay

The combination of Goldenrod herb and antibiotics was assessed using the checkerboard assay, as outlined in the study conducted by Berditsch et al. (2015) [38]. To evaluate the effects of the SV extract and antibiotics in combination, a 96-well microtiter plate was utilized. The SV extract and antibiotics were diluted in two-fold increments, with the SV extract diluted horizontally and the antibiotics diluted vertically, starting from their respective minimum inhibitory concentrations (MICs). Each well contained 100 μL of either the SV extract alone or a combination of SV extract and antibiotics, along with 100 μL of a bacterial suspension (at a concentration of 1 × 10^5^ CFU/mL). Negative controls consisted of MH medium while positive controls included the SV extract or antibiotics used individually. Following overnight incubation at 37 °C, the optical density at 600 nm (OD_600_) was measured using an ELISA microplate reader (Thomas scientific, Swedesboro, NJ, USA). Synergistic interactions were assessed using the fractional inhibitory concentration index (FICI), calculated as the sum of FICa and FICb. FICa represents the ratio of the MICs of the SV extract in combination to the MICs of the SV extract alone while FICb represented the ratio of the MICs of the antibiotics in combination to the MICs of the antibiotics alone. Synergy, addition and indifference were defined as FICI values of ≤0.5, 0.5 < FICI ≤ 1.0 and 1.0 < FICI ≤ 2.0, respectively [39].

### 4.13. Analysis of Research Data

Graph pad prism (version: 8.0) software was used for statistical analysis. Chi-square and paired *t*-tests were applied. A *p*-value less than 0.05 was considered statistically significant.

## 5. Conclusions

The results obtained from this initial investigation are noteworthy and innovative, as they highlight the capability of the goldenrod herb extract to inhibit the growth of planktonic forms of *Pseudomonas aeruginosa* and hinder the formation of biofilms during the early stages of a culture. Furthermore, the extract was found to effectively inhibit the formation of extended-spectrum β-lactamases (ESBLs). It is crucial to implement active surveillance not only to monitor antimicrobial resistance, but also to track ESBLs and biofilm factors. This is essential in order to prevent the dissemination and proliferation of pan drug-resistant *P. aeruginosa* strains.

## Figures and Tables

**Table 1:** Confirmation tests of *P. aeruginosa*.

No.	Test	*P. aeruginosa*
1	MacConkey agar medium	Non-lactose fermented
2	Blood Agar	β-hemolysis
3	Chromogenic agar	Blue colonies
4	Gram-stain	G-rod
5	Catalase test	Positive
6	Oxidase test	Positive
7	Indol Test	Negative
8	Methyl Red Test	Negative
9	VP Test	Negative
10	Citrate Utilization Test	Positive
11	Urease	Negative

**Table 2:** Analysis of SV extract using GC–MS.

Peak	Active Compound	Area%	^a^ RT (min)	^b^ RRI
1	Ethanamine	0.99	4.632	4663
2	Malic Acid	1.02	5.320	14,971
3	Silver acetate	0.71	5.685	35,237
4	1,6-Hexanediamine	0.58	8.251	8254
5	Cytisine	0.55	14.393	52,877
6	Lysicamine monophenol	3.04	15.837	125,087
7	Acetamide	1.34	17.018	20,526
8	5-Acetoxytridecane	1.39	17.341	95,848
9	1,9-Diaminononane	0.63	19.091	30,366
10	2,4(1H,3H)-Pyrimidinedione	0.63	20.229	20,528
11	Clioquinol	5.77	21.512	148,956
12	Propanamide	20.29	22.242	49,432
13	Oleic Acid	1.89	22.973	129,336
14	Bromoacetic acid	1.63	23.984	207,396
15	trans-13-Octadecenoic acid	0.71	27.025	129,357
16	2-Methyl-Z,Z-3,13-octadecadienol	1.76	28.138	127,747
17	Oleic Acid	2.19	29.582	129,336
18	Octadecane	45.21	30.653	194,511
19	Fumaric acid	0.76	32.352	194,365
20	Glycerol tricaprylate	8.92	33.873	231,044
Total	100%			

^a^ RT: retention time; ^b^ RRI: Relative Retention Indices.

**Table 3:** MIC Values of Antibiotics and SV Extracts Against *P. aeruginosa*.

Sr. No.	Bacterial Strain	Concentration of Antibiotic at Which MIC Was Obtained (µg mL^−1^)			Concentration of Crude Plant Extracts at Which MIC Was Obtained (mg mL^−1^)
		Ceftazidime	Cefepime	Amikacin	SA Extract
1	P1	128	32	32	64
2	P2	64	64	16	16
3	P3	64	32	32	0.25
4	S1	16	16	8	32
5	S2	32	8	16	8

Sr. No.: Strain nomber.

**Table 4:** Effect of SV extract against biofilm and ESBL producing *P. aeruginosa*.

Name	A: Before Treatment with SV Extract M ± SD	B: After Treatment with SV Extract M ± SD	*p*-Value
ESBL activity (nitrocefin hydrolyzed/min/mg)	0.4447 ± 0.2793	0.5812 ± 0.2837	0.0001 ***
Biofilm assay (at OD630nm)	0.1028 ± 0.05215	0.05515 ± 0.02532	0.0004 ***

M: Mean; SD: Std. Deviation; ***: strong significant.

**Table 5:** Correlation between ESBL and biofilm formation among *P. aeruginosa* isolates.

Type	ESBL Producing (n:36)	Non-ESBL Producing (n:14)	Total	*p*-Value
Strong biofilm	20 (55.5)	5 (35.7)	25	<0.05
Moderate biofilm	8 (22.8)	2 (14.2)	10	
Weak biofilm	2 (5.5)	3 (21.4)	5	
Non-biofilm	6 (16.6)	4 (28.5)	10	
Total	36	14	50	

**Table 6:** Antimicrobial agents combination between SV extract with some antibiotics.

FICI				
Plant	Ceftazidime	Cefepime	Amikacin	Outcome
SV extract	**0.91**	**0.61**	**0.664**	Additive

## Data Availability

Data is contained within the article.

## References

[1] Al Maeni S.A.L., Ahmed M.M., Abed A.D., Abbas R.H., Jassim S.A.A., Mohamad A.M. (2021). Extraction and Purification of Staphylolysin Enzyme from Local Isolate of *Pseudomonas aeruginosa*. Ann. Rom. Soc. Cell Biol..

[2] Rossi E., La Rosa R., Bartell J.A., Marvig R.L., Haagensen J.A.J., Sommer L.M., Molin S., Johansen H.K. (2021). *Pseudomonas aeruginosa* adaptation and evolution in patients with cystic fibrosis. Nat. Rev. Microbiol..

[3] Seder N., Rayyan W.A., Al-Fawares M.H.O., Bakar A. (2023). *Pseudomonas aeruginosa* Virulence Factors and Antivirulence mechanisms to Combat Drug Resistance; A Systematic Review. Mortality.

[4] Darby E.M., Trampari E., Siasat P., Gaya M.S., Alav I., Webber M.A., Blair J.M.A. (2023). Molecular mechanisms of antibiotic resistance revisited. Nat. Rev. Microbiol..

[5] Qin S., Xiao W., Zhou C., Pu Q., Deng X., Lan L., Liang H., Song X., Wu M. (2022). *Pseudomonas aeruginosa*: Pathogenesis, virulence factors, antibiotic resistance, interaction with host, technology advances and emerging therapeutics. Signal Transduct. Target. Ther..

[6] Shaikh S., Fatima J., Shakil S., Rizvi S.M.D., Kamal M.A. (2015). Antibiotic resistance and extended spectrum beta-lactamases: Types, epidemiology and treatment. Saudi J. Biol. Sci..

[7] Teklu D.S., Negeri A.A., Legese M.H., Bedada T.L., Woldemariam H.K., Tullu K.D. (2019). Extended-spectrum beta-lactamase production and multi-drug resistance among *Enterobacteriaceae* isolated in Addis Ababa, Ethiopia. Antimicrob. Resist. Infect. Control.

[8] Ahmed M.M., Khadum H.E., Jassam H.M.S. (2023). Medicinal Herbs as Novel Therapies against Antibiotic-Resistant Bacteria. Res. J. Pharm. Technol..

[9] Hussein M.S., Al-Qaysi A.-M.K., Al Meani S.A.L., Ahmed M.M., Abed N.S., Ibrahim M.O. (2022). Assessment of Cinamic acid and Costus roots extract against MDR-*K. pneumoniae* isolated from patients of COVID-19. Res. J. Pharm. Technol..

[10] Fursenco C., Calalb T., Uncu L., Dinu M., Ancuceanu R. (2020). *Solidago virgaurea* L.: A review of its ethnomedicinal uses, phytochemistry, and pharmacological activities. Biomolecules.

[11] Móricz A.M., Ott P.G., Häbe T.T., Darcsi A., Böszörményi A., Alberti A., Krüzselyi D., Csontos P., Béni S., Morlock G.E. (2016). Effect-directed discovery of bioactive compounds followed by highly targeted characterization, isolation and identification, exemplarily shown for *Solidago virgaurea*. Anal. Chem..

[12] Chevalier M., Doglio A., Rajendran R., Ramage G., Prêcheur I., Ranque S. (2019). Inhibition of adhesion-specific genes by *Solidago virgaurea* extract causes loss of *Candida albicans* biofilm integrity. J. Appl. Microbiol..

[13] Rosłon W., Osińska E., Mazur K., Geszprych A. (2014). Chemical characteristics of European goldenrod (*Solidago virgaurea* L. subsp. *virgaurea*) from natural sites in Central and Eastern Poland. Acta Sci. Pol. Hortorum Cultus.

[14] Akinduti P.A., George O.W., Ohore H.U., Ariyo O.E., Popoola S.T., Adeleye A.I., Akinwande K.S., Popoola J.O., Rotimi S.O., Olufemi F.O. (2023). Evaluation of Efflux-Mediated Resistance and Biofilm formation in Virulent *Pseudomonas aeruginosa* Associated with Healthcare Infections. Antibiotics.

[15] Kamali E., Jamali A., Ardebili A., Ezadi F., Mohebbi A. (2020). Evaluation of antimicrobial resistance, biofilm forming potential, and the presence of biofilm-related genes among clinical isolates of *Pseudomonas aeruginosa*. BMC Res. Notes.

[16] Vaez H., Salehi-Abargouei A., Ghalehnoo Z.R., Khademi F. (2018). Multidrug resistant *Pseudomonas aeruginosa* in Iran: A systematic review and metaanalysis. J. Glob. Infect. Dis..

[17] Bavasheh N., Karmostaji A. (2017). Antibiotic resistance pattern and evaluation of blaOXA-10, blaPER-1, blaVEB, blaSHV genes in clinical isolates of *Pseudomonas aeruginosa* isolated from hospital in south of Iran in 2014-2015. Infect. Epidemiol. Microbiol..

[18] Faghri J., Nouri S., Jalalifar S., Zalipoor M., Halaji M. (2018). Investigation of antimicrobial susceptibility, class I and II integrons among *Pseudomonas aeruginosa* isolates from hospitalized patients in Isfahan, Iran. BMC Res. Notes.

[19] Ölgen S., Altanlar N., Karataylı E., Bozdayı M. (2008). Antimicrobial and antiviral screening of novel indole carboxamide and propanamide derivatives. Z. Naturforsch. C.

[20] Chaudhary S., Verma H.C., Gupta M.K., Gupta R.K., Kumar A., El-Shorbagi A.N. (2019). Synthesis and investigation of anthelmintic, antibacterial and antifungal activity of 3, 3-diphenyl propanamide derivatives. Synthesis.

[21] Yasir A., Jahangir M., Ishtiaq S., Shahid M. (2017). Antimicrobial, hemolytic and thrombolytic activities of some new N-substituted-2-({5-[(1E, 3E) F-4-(1, 3-benzodioxol-5-yl)-1, 3-butadienyl]-1, 3, 4-oxadiazol-2-yl} sulfanyl) propanamides. Trop. J. Pharm. Res..

[22] You-Qing L., Hong-Fang L., Zhen-Le T., Li-Hua Z., Ying-Hui W., He-Qing T. (2008). Diesel pollution biodegradation: Synergetic effect of Mycobacterium and filamentous fungi. Biomed. Environ. Sci..

[23] Blanco P., Corona F., Martinez J.L. (2018). Biolog phenotype microarray is a tool for the identification of multidrug resistance efflux pump inducers. Antimicrob. Agents Chemother..

[24] De Majumdar S., Veleba M., Finn S., Fanning S., Schneiders T. (2013). Elucidating the regulon of multidrug resistance regulator RarA in *Klebsiella pneumoniae*. Antimicrob. Agents Chemother..

[25] Chevalier M., Medioni E., Prêcheur I. (2012). Inhibition of Candida albicans yeast–hyphal transition and biofilm formation by *Solidago virgaurea* water extracts. J. Med. Microbiol..

[26] Balázs V.L., Nagy-Radványi L., Bencsik-Kerekes E., Koloh R., Szabó D., Kocsis B., Kocsis M., Farkas Á. (2023). Antibacterial and Antibiofilm Effect of Unifloral Honeys against Bacteria Isolated from Chronic Wound Infections. Microorganisms.

[27] Maia N.L., de Barros M., de Oliveira L.L., Cardoso S.A., dos Santos M.H., Pieri F.A., Ramalho T.C., da Cunha E.F.F., Moreira M.A.S. (2018). Synergism of plant compound with traditional antimicrobials against *Streptococcus* spp. isolated from bovine mastitis. Front. Microbiol..

[28] Wojnicz D., Tichaczek-Goska D., Gleńsk M., Hendrich A.B. (2021). Is it Worth Combining *Solidago virgaurea* Extract and Antibiotics against Uropathogenic *Escherichia coli* rods? An *In Vitro* Model Study. Pharmaceutics.

[29] Finegold S.M., Martin W.J. (1982). Bailey and Scotts diagnostic microbiology. Bailey and Scotts Diagnostic Microbiology.

[30] Clinical And Laboratory Standars Institute (2018). M100 Performance Standards for Antimicrobial Susceptibility Testing.

[31] (2020). Committee, The European Testing, Antimicrobial Susceptibility Changes, Content Guidance, Notes Breakpoint, Eu-cast Dosages, Tables Pseudomonas, Enterobacterales Document, Guidance Document, Guidance. European Committee on Anti-microbial Susceptibility Testing Breakpoint Tables for Interpretation of MICs and Zone Diameters. https://www.eucast.org/.

[32] Bauer A.W., Kirby W.M.M., Sherris J.C., Turck M. (1966). Antibiotic susceptibility testing by a standardized single disk method. Am. J. Clin. Pathol..

[33] Kebede B., Shibeshi W. (2022). *In vitro* antibacterial and antifungal activities of extracts and fractions of leaves of *Ricinus communis* Linn against selected pathogens. Vet. Med. Sci..

[34] Freeman D.J., Falkiner F.R., Keane C.T. (1989). New method for detecting slime production by coagulase negative staphylococci. J. Clin. Pathol..

[35] O’Toole G.A., Kolter R. (1998). Initiation of biofilm formation in *Pseudomonas fluorescens* WCS365 proceeds via multiple, convergent signalling pathways: A genetic analysis. Mol. Microbiol..

[36] Kong W., Chen L., Zhao J., Shen T., Surette M.G., Shen L., Duan K. (2013). Hybrid sensor kinase PA 1611 in *Pseudomonas aeruginosa* regulates transitions between acute and chronic infection through direct interaction with RetS. Mol. Microbiol..

[37] Pérez-Llarena F., Martín J.F., Galleni M., Coque J.J., Fuente J.L., Frère J.M., Liras P. (1997). The bla gene of the cephamycin cluster of *Streptomyces clavuligerus* encodes a class A beta-lactamase of low enzymatic activity. J. Bacteriol..

[38] Berditsch M., Jäger T., Strempel N., Schwartz T., Overhage J., Ulrich A.S. (2015). Synergistic effect of membrane-active peptides polymyxin B and gramicidin S on multidrug-resistant strains and biofilms of *Pseudomonas aeruginosa*. Antimicrob. Agents Chemother..

[39] den Hollander J.G., Mouton J.W., Verbrugh H.A. (1998). Use of pharmacodynamic parameters to predict efficacy of combination therapy by using fractional inhibitory concentration kinetics. Antimicrob. Agents Chemother..

